# A phase II feasibility study of carboplatin followed by sequential weekly paclitaxel and gemcitabine as first-line treatment for ovarian cancer

**DOI:** 10.1038/sj.bjc.6602000

**Published:** 2004-07-06

**Authors:** M Harries, C Moss, T Perren, M Gore, G Hall, M Everard, R A'Hern, I Gibbens, A Jenkins, R Shah, C Cole, O Pizzada, S Kaye

**Affiliations:** 1 Section of Medicine, Institute of Cancer Research and Royal Marsden Hospital, Downs Road, Sutton, Surrey, SM2 5PT, London, UK; 2 Cancer Research UK Clinical Centre, St James's University Hospital, Leeds, UK

**Keywords:** feasibility, carboplatin, sequential paclitaxel, gemcitabine ovarian cancer

## Abstract

A total of 53 women with chemotherapy-naïve stage Ic-IV ovarian cancer were treated with four cycles of carboplatin area under the curve 7 every 3 weeks, followed by four cycles of paclitaxel 70 mg m^−2^ (days 1, 8, and 15) and gemcitabine 1000 mg m^−2^ (days 1 and 8) every 3 weeks. In all, 37 (70%) had stage III/IV disease, with 22 (42%) having tumour >2 cm; 38 patients (72%) completed all planned treatment; 27 of the 32 (84%) patients with radiologically evaluable disease had partial or complete responses; and 30 of the 39 patients (77%) with elevated cancer antigen (CA) 125 had a greater than 75% fall in this value. At a median follow-up of 28 months, 31 patients had relapsed with a median progression-free survival of 19.5 months. In total, 79% of patients were alive at 2 years. Common Toxicity Criteria grade 3/4 haematological toxicity, predominantly neutropenia, was seen in 57% of the patients. A certain degree of pulmonary toxicity was observed; eight patients had symptomatic breathlessness, ± decreased diffusing capacity of the lung for carbon monoxide, and interstitial chest X-ray changes during the weekly phase. In all cases, this toxicity was reversible. No significant neurotoxicity was seen. This regimen is generally well tolerated with encouraging efficacy. However, the observation of pulmonary toxicity, potentially a feature of the weekly taxane–gemcitabine regimen, was of some concern. Alternative schedules, including 3-weekly taxanes, are currently being evaluated.

The majority of patients with epithelial ovarian cancer will require chemotherapy. It is now well established that first-line treatment should include a platinum compound (Harries and Gore, 2002), with carboplatin being the drug of choice, having been shown to have equivalent efficacy but less toxicity than cisplatin (Alberts, 1995; Du Bois *et al*, 1999; Ozols *et al*, 2003). Two large randomised studies in the 1990s of first-line therapy for ovarian cancer showed a survival benefit when a paclitaxel–platinum combination was compared with the previous standard of cyclophosphamide–cisplatin; therefore, the combination of platinum and paclitaxel became standard first-line therapy (McGuire *et al*, 1996; Piccart *et al*, 2000). Two other large randomised trials conducted by the International Collaborative Ovarian Neoplasm (ICON) group (ICON group, 2002) and the Gynaecologic Oncology Group (GOG) (Muggia *et al*, 2000) failed to confirm the benefit of the addition of paclitaxel to platinum. The reasons are unclear, but a negative interaction between the two drugs is not excluded. It is interesting to note that taxanes, when given in short infusions, clearly reduce the thrombocytopenia expected from carboplatin; again the mechanism is not understood. A number of patients in the non-taxane-containing arms of the ICON and GOG trials crossed over to receive paclitaxel at disease progression, and since results were equivalent this suggests that there may be no disadvantage to giving platinum and taxanes sequentially rather than concurrently. The ICON group has recently shown a benefit when paclitaxel was added to platinum in patients with potentially platinum-sensitive relapsed disease (Parmar *et al*, 2003).

Despite the benefits observed in the above trials, however, the majority of women in these studies eventually died from their disease, thus underscoring the importance of designing trials to improve the results achieved with the combination of paclitaxel and platinum. One approach is to add other potentially non-cross-resistant agents and drugs that are being evaluated in this setting include topotecan, liposomal doxorubicin, and gemcitabine.

Gemcitabine, a pyrimidine analogue, is active in untreated and pretreated ovarian cancer. In a small study of chemonaive patients, single-agent gemcitabine produced a modest overall response rate of 18% (D'Agostino *et al*, 2003). In patients with platinum-pretreated, relapsed ovarian cancer, response rates ranged from 13 to 28%, which are comparable with those of other agents (e.g., topotecan, liposomal doxorubicin, and paclitaxel) in a similar patient population (Lund *et al*, 1994; Shapiro *et al*, 1996; Friedlander *et al*, 1998; Silver and Piver, 1999; von Minckwitz *et al*, 1999; Underhill *et al*, 2001). Gemcitabine is generally well tolerated with common toxicities including myelosuppression, lethargy, flu-like symptoms, peripheral oedema, and skin rashes. An unexplained rare severe pulmonary toxicity has been reported, particularly in patients with lung cancer who have received prior mediastinal radiotherapy (Sauer-Heilborn *et al*, 1999).

Gemcitabine in combination with platinum and paclitaxel as a triplet regimen has been explored recently as first-line therapy for ovarian cancer (Hansen *et al*, 1999; Hansen, 2002). Although the response rate was highly favourable (overall response rate of 100% in 25 evaluable patients), haematological toxicity was significant, with grade 4 myelosuppression reported in 96% of patients.

In the trial reported here, we evaluated the addition of gemcitabine to carboplatin and paclitaxel in a sequential regimen, with carboplatin monotherapy followed by gemcitabine and paclitaxel to chemonaive patients with ovarian cancer. A sequential regimen in which carboplatin is given first and separately from paclitaxel may reduce toxicity as well as decrease the possibility of any antagonism between these agents. It might also take advantage of the laboratory observation that the presence of p53 mutant cells determines the relative efficacy of carboplatin and paclitaxel; sequential treatment could theoretically be more effective through activity on two subpopulations of tumour cells, one after the other (Cassinelli *et al*, 2001). The feasibility of the regimen (i.e., the ability to deliver the planned doses and its toxicity) and its efficacy (response rates, cancer antigen (CA) 125 response rates, and progression-free survival) are reported here.

## PATIENTS AND METHODS

### Eligibility criteria

Women aged 18 or older with chemotherapy-naïve histologically confirmed epithelial ovarian, primary peritoneal, or fallopian tube carcinomas were eligible for study entry. Patients had International Federation of Gynaecology and Obstetrics (FIGO) stages Ic-IV disease with or without successful cytoreductive surgery. Patients with Ic disease were limited to those with malignant cells in ascitic fluid, peritoneal washings or with tumour on the surface of the ovary. Patients had to be within 8 weeks of initial laparotomy/surgery and able to comply with follow-up requirements. Each patient had to have an Eastern Cooperative Oncology Group (ECOG) performance status of 0, 1, or 2. Adequate liver (bilirubin ⩽upper limit of normal (ULN), aspartate aminotransferase (AST)/alanine aminotransferase (ALT) ⩽1.5 × ULN, or alkaline phosphatase (ALP) ⩽3 × ULN), renal (creatinine ⩽1.25 × ULN), and bone marrow functions (neutrophils ⩾1.5 × 10^9^ l^−1^, platelets ⩾100 × 10^9^ l^−1^, and haemoglobin ⩾9.0 g l^−1^) were required.

Patients were excluded from the study if they had borderline ovarian malignancies, ruptured capsule as the only evidence of stage Ic, mixed mesodermal tumours, a previous malignancy (except for *in situ* cervical cancer or non-melanoma skin cancer), concurrent severe and/or uncontrolled co-morbid medical conditions, history of prior serious allergic reactions (ie anaphylaxis), or symptomatic peripheral neuropathy ⩾grade 2 (National Cancer Institute of Canada-Common Toxicity Criteria (CTC)). Other exclusion criteria included: history of insulin-dependent diabetes mellitus or other relative contraindications to corticosteroids or pregnant or lactating women (or potentially fertile women not using adequate contraception). The study was approved by the local ethics committees, and written informed consent was obtained from all patients before study entry.

### Treatment plan

The chemotherapy regimen consisted of a total of eight cycles, with four cycles of carboplatin area under the curve (AUC) 7 (glomerular filtration rate (GFR) calculated using ^51^Cr EDTA clearance) given at 3-week intervals, followed by four cycles of paclitaxel 70 mg m^−2^ on days 1, 8, and 15 and gemcitabine 1000 mg m^−2^ on days 1 and 8 at 3-week intervals. Carboplatin was given as a 1-h infusion in 500 ml 5% glucose. After completing carboplatin therapy, interval debulking surgery was considered for those patients who had not undergone optimal debulking at their first operation. Paclitaxel was given as a 1-h infusion in 250 ml 5% glucose, followed by gemcitabine as a 30-min infusion in 250 ml 0.9% saline. Standard premedications, such as dexamethasone and 5HT_3_ antagonists, were used.

Full doses of carboplatin were given if neutrophils were ⩾1.5 × 10^9^ l^−1^ and platelets were ⩾100 × 10^9^ l^−1^ on the day of treatment. If, on the day of treatment, neutrophil and platelet levels were not within these ranges, carboplatin was delayed for up to 2 weeks. A delay of >2 weeks for haematological recovery necessitated withdrawal from study treatment. If the blood count had recovered between 1 and 2 weeks or if prolonged neutropenia, neutropenic sepsis, or complicated grade 4 thrombocytopenia occurred, the subsequent dose of carboplatin was reduced by 1 AUC level. If the toxicity persisted, the carboplatin dose could only be reduced to AUC 5 before withdrawal of the patient from the study.

During weekly paclitaxel/gemcitabine treatment, the same minimum threshold for a treatment day blood count was employed as above. However, after several patients had experienced dose delays, it was decided to amend the threshold for an acceptable neutrophil count. Thus, on each treatment day (days 1, 8, and 15), full doses of all drug(s) were given if neutrophils were ⩾1.0 × 10^9^ l^−1^ and platelets were ⩾100 × 10^9^ l^−1^. If these levels were not reached, treatment was delayed for up to 2 weeks. A delay of more than 2 weeks for haematological recovery necessitated removal from study treatment. If blood counts recovered between 1 and 2 weeks or if neutropenic sepsis or complicated grade 4 thrombocytopenia occurred, the subsequent doses of both drugs were reduced by 25%. If grade 3/4 mucositis (or at physician's discretion if grade 2) or ⩾grade 2 skin toxicity occurred, treatment was delayed. For subsequent cycles, paclitaxel/gemcitabine doses were reduced by 25%. Treatment was stopped if grade 3/4 neurotoxicity or ototoxicity occurred. If hypersensitivity reactions to paclitaxel occurred, the infusion was stopped immediately and treated accordingly, or restarted after recovery at the discretion of the attending physician. If the appearance of any intrapulmonary infiltrates and/or the development of any unexplained pulmonary symptoms such as dyspnoea, wheezing, or cough occurred, treatment was delayed until pathogenesis could be determined. If this was drug-related, the patient did not receive further therapy.

### Baseline and treatment assessments

Prior to study entry, each patient underwent a standard physical examination (including neurological and pelvic examination) with an electrocardiogram and chest X-ray. Disease state via radiological assessment per abdominal and pelvic computed tomography (CT), ECOG performance status, weight, and GFR were also assessed at baseline.

Safety assessments were conducted at the start of treatment and included full blood count (FBC) including differential white cell count and full biochemical profile (urea, creatinine, sodium, potassium, calcium, magnesium, AST, ALT, ALP, bilirubin, total protein, albumen, and glucose). Glomerular filtration rate was measured using ^51^Cr EDTA. The FBC and biochemical profile were performed on day 1 of carboplatin therapy and weekly during paclitaxel and gemcitabine therapy.

Before each cycle of therapy, a standard physical exam was performed, and toxicity, performance status, and weight were reassessed. Chest X-rays were repeated during the carboplatin cycles if disease was evident at baseline or if clinically indicated. An additional CT scan and chest X-ray were performed before and after the four cycles of paclitaxel and gemcitabine if evaluable disease was evident at baseline, if progressive disease occurred, or if the patient underwent interval debulking. A physical examination and neurotoxicity assessment were also performed, before and after the four cycles of paclitaxel and gemcitabine. All patients received a chest X-ray prior to each cycle of paclitaxel and gemcitabine therapy.

Clinical response was determined by CT scan in patients with measurable disease at the start of chemotherapy. If the CT scan showed evaluable disease, patients were deemed evaluable for response. Scans were performed at baseline and repeated after the four cycles of carboplatin (both before and after surgery if interval debulking was performed) and once all eight cycles of chemotherapy were completed. Standard WHO clinical response criteria were used.

CA 125 levels were measured before each cycle of chemotherapy. For the patients with an elevated CA 125 at the start of therapy, the proportions of patients with CA 125 reductions >75 and 50% from baseline were determined after the four cycles of carboplatin (and before any interval debulking) and again at the completion of chemotherapy.

Disease progression was defined by radiological findings or by a rising CA 125 in association with symptoms. CA 125 elevation alone, in the absence of clinical evidence of relapse, was not sufficient evidence for progression. Progression-free survival for all patients was recorded from the date of the first cycle of chemotherapy until disease progression or death from any cause, and overall survival from the date of the first cycle of chemotherapy until death from any cause. Kaplan–Meier progression-free and overall survival curves were generated.

### Statistical considerations

This was a phase II trial conducted at two cancer centres in the United Kingdom. The trial was designed to assess whether the experimental regimen was feasible as determined by the proportion of patients completing all the eight cycles of chemotherapy. The number of patients required for study entry was based on the premise that ⩾80% is clearly an acceptable study completion rate, 60–80% is a ‘grey area,’ and ⩽60% is clearly unacceptable. The study was designed to test the null hypothesis that the completion rate is ⩽60% against the alternative that it is >60%. The one-sided significance level was set at 5% and the power of the study for a true completion rate of 80% was set at 90%. This necessitated a target enrolment of 44 patients.

## RESULTS

### Patient characteristics

Between January 2001 and January 2002, 53 patients were enrolled into the study. The majority of patients had advanced stage ovarian cancer, with six patients (11%) having stage IV and 31 (58%) stage III disease (Table 1

**Table 1:** Baseline characteristics of patients (*n*=53)

*Age (years)*	
Median (range)	**59 (29–77)**
	
*FIGO stage*	
Ic	11 (21%)
II	5 (9%)
III	31 (58%)
IV	6 (11%)
CA 125 elevated	39 (74%)
	
*Histology*	
Serous/papillary	27 (51%)
Endometroid	11 (21%)
Clear cell	3 (6%)
Mucinous	3 (6%)
Other	8 (15%)
Unknown	1 (2%)
	
*Residual disease*	
Microscopic	20 (38%)
⩽2 cm	11 (21%)
>2 cm	22 (42%)

FIGO=International Federation of Gynaecology and Obstetrics.

). In all, 20 patients had no residual macroscopic disease after surgery, 11 patients had 2 cm or less, and 22 patients (42%) had >2 cm of residual disease prior to chemotherapy. A total of 39 patients (74%) had an elevated CA 125 at baseline and 32 (60%) had radiologically evaluable disease. The majority of patients (72%) had either serous/papillary or endometroid histological subtypes of ovarian cancer.

### Drug administration

A total of 38 patients (72%) completed all eight cycles of treatment. In all, 15 patients did not complete the full course of therapy; four due to disease progression, 10 due to toxicities, and one due to patient choice. Patients completed a total of 203 cycles of carboplatin and a 167 cycles of paclitaxel/gemcitabine, for a total of 370 cycles completed. Doses were reduced in 12 carboplatin cycles (6%) and 15 paclitaxel/gemcitabine cycles (9%). Doses were delayed in 37 carboplatin cycles (18%) due to lack of haematologic recovery by day 21 and in 19% of paclitaxel/gemcitabine cycles primarily due to myelosuppression.

### Toxicity

Neutropaenia was the major haematological toxicity encountered for the 51 patients evaluable for toxicity (two patients who developed disease progression during the second cycle of carboplatin were not considered evaluable for toxicity) (Table 2

**Table 2:** Worst grade 3/4 haematological toxicity by patient (*n*=51)

	**Carboplatin**		**Paclitaxel/Gemcitabine**	
	**Grade 3 (%)**	**Grade 4 (%)**	**Grade 3 (%)**	**Grade 4 (%)**
Anaemia	(4)	(2)	0	0
Thrombocytopenia	(10)	(8)	(4)	(7)
Neutropenia	(20)	(37)	(28)	(57)

). Grade 4 neutropenia occurred in 37% of patients during carboplatin therapy and 57% of patients during paclitaxel/gemcitabine therapy. No episodes of febrile neutropenia were reported during the conduct of the study. Thrombocytopenia was infrequent, with 18 and 11% of patients developing grade 3/4 thrombocytopenia with carboplatin and paclitaxel/gemcitabine, respectively, and was not associated with any clinical complications.

During carboplatin therapy, grade 3 nausea and vomiting was the most common nonhaematological toxicity, occurring in 10% of patients (Table 3

**Table 3:** Worst grade nonhaematological toxicity by patient (*n*=51)

	**Carboplatin**	**Paclitaxel/Gemcitabine**
	**Grade 3^a^**	**Grade 3^a^**
Alopecia	0	0
Nausea/vomiting	(10)	0
Neuropathy	(2)	0
Diarrhoea	0	(2)
Constipation	0	(2)
Stomatitis	(2)	0
Infection	0	(4)
Lethargy/fatigue	(2)	(8)
Anxiety	0	(2)
Pulmonary	(2)	(2)

a No grade 4 nonhaematological toxicity was seen.

). The predominant grade 3/4 nonhaematological toxicities for paclitaxel/gemcitabine treatment were lethargy/fatigue (8%) and infection (4%). Alopecia, nausea and vomiting, and neuropathy occurred infrequently during the conduct of the study.

Several patients developed respiratory symptoms and, as a consequence, pulmonary toxicity was examined in more detail. One patient (2%) developed pneumonia while receiving carboplatin; this was thought to be unrelated to chemotherapy. In all, 10 patients had some degree of symptomatic pulmonary toxicity during treatment with gemcitabine/paclitaxel. Two patients developed breathlessness that was thought to be unrelated to treatment (one had an exacerbation of asthma diagnosed by a respiratory physician and another a Haemophilus Influenza pneumonia). Eight developed symptoms of dyspnoea (three grade 3 and five grade 2) that were considered related to chemotherapy with or without decreased DLCO and radiological interstitial lung changes. Four developed symptoms in cycle five, one in cycle 6, one in cycle 7 and two in cycle 8. These events are now described.

One woman developed shortness of breath at rest in cycle 7 (CTC grade 3) associated with pulmonary infiltrates on chest X-ray and decreased DLCO. She received corticosteroids, but subsequently developed disease progression with pleural effusions. Two patients developed acute onset grade 3 dyspnoea and cough in cycle 5, their symptoms resolved in 1 week and no further gemcitabine was given. Five patients developed shortness of breath on exertion only (CTC grade 2). Two of these patients developed symptoms in cycle 5; one was given corticosteroids and in both cases toxicity was reversible and short lived. Three further patients developed CTC grade 2 dyspnoea. One patient who developed symptoms in cycle 6 developed further respiratory problems caused by multiple pulmonary emboli 3 months later. The two other patients developed grade 2 dyspnoea in cycle 8, which resolved in 4 weeks and 4 months, respectively.

### Efficacy

After the four carboplatin cycles and before any further surgery, 10 of the 32 patients (31%) with measurable disease achieved a complete response (CR) and 16 patients (50%) had a partial response (PR) for a response rate of 81% (Table 4

**Table 4:** Response assessment

	**Post carboplatin**	**Overall**
*WHO response* (*n*=32)		
CR	10 (31%)	13 (40%)
PR	16 (50%)	14 (44%)
CR+PR	26 (81%)	27 (84%)
SD	4 (13%)	1 (3%)
PD	2 (6%)	4 (13%)

*CA 125 response (n*=*39*)		
>75% decrease	28 (72%)	30 (77%)
>50% decrease	32 (82%)	32 (82%)
<50% decrease	2 (5%)	2 (5%)
Increase	2 (5%)	2 (5%)
Unknown	3 (8%)	3 (8%)

WHO=World Health Organisation; CR=complete response; PR=partial response; SD=stable disease; PD=progressive disease; CA=cancer antigen.

). Stable disease (SD) was reported in four patients (16%) and two patients (6%) progressed. Five patients (9%) underwent interval debulking surgery, two of whom had complete resection of all macroscopic disease. On the subsequent weekly paclitaxel/gemcitabine regimen, three of the 16 patients with PR achieved CR, and a patient with SD achieved PR. Thus, the overall response rate was 84% (CR 13 out of 32, PR 14 out of 32). Of the remaining 21 patients with non-measurable or non-evaluable disease, none had progressed at the end of therapy. Of the three patients with clear cell histology, two had evaluable disease and both achieved CR. Of the three patients with mucinous histology, two had nonevaluable disease and one developed PD.

Of the 39 patients with elevated CA 125 at baseline, 82% had a >50% decrease in CA 125 and 72% had a >75% decrease after four cycles of carboplatin. At the end of chemotherapy, these rates were 82 and 77%, respectively (Table 4).

**Figure 1:**
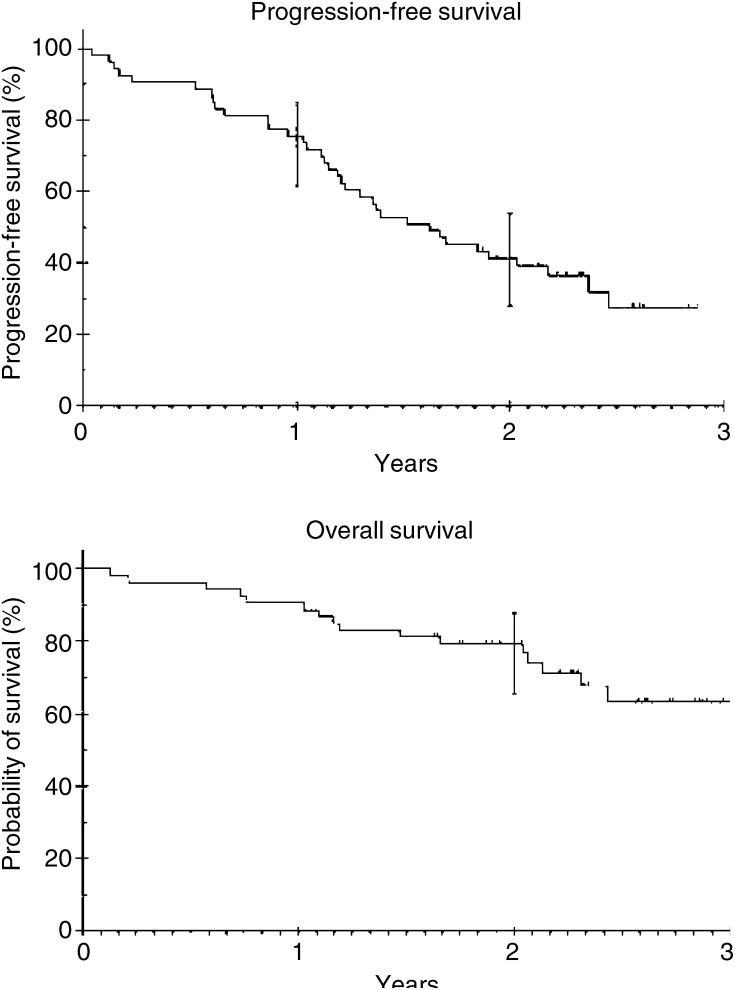
Kaplan–Meier analysis of PFS and overall survival (OS) of all patients. Median follow-up 28 months. Median PFS 19.5 months (95% CI: 13.4–25.6 months). PFS at years 1 and 2 and OS at year 2 are indicated with error bars showing 95% confidence intervals.

As of March 2004, with a median follow-up of 28 months, 31 patients had relapsed. The median progression-free survival was 19.5 months (95% CI: 13.4–25.6 months, Figure 1

). Median overall survival has not yet been reached, and 79.2% of patients were alive at 2 years (95% CI: 65.5–87.9%).

## DISCUSSION

The current study is part of a portfolio of studies conducted by the Scottish Gynaecological Cancer Trials Group (SGCTG) to examine the feasibility of sequential chemotherapy scheduling. The schedule chosen for this study mirrored the weekly arm of the SGCTG Scottish Randomised Trial in Ovarian Cancer (SCOTROC) 2a trial, in which patients received four cycles of carboplatin AUC 7 every 3 weeks, followed by four cycles of docetaxel 25 mg m^−2^ and gemcitabine 800 mg m^−2^, both drugs given on days 1, 8, and 15 every 3 weeks (Vasey *et al*, 2003). Although the SGCTG has recently demonstrated that docetaxel has equivalent efficacy to paclitaxel in first-line therapy (Vasey, 2001, 2002), we used the more established taxane in ovarian cancer, paclitaxel.

Our study demonstrated that sequential treatment with four cycles of carboplatin AUC 7 followed by paclitaxel and gemcitabine is feasible as primary treatment for advanced ovarian cancer with 72% of patients being able to complete all the eight cycles of treatment. This is an active regimen with an overall radiological response rate of 84% and >75% decrease in CA 125 seen in 72% of patients. In all, 37 out of 53 (70%) patients had initial stage III or IV disease, of whom 15 (40%) had been optimally debulked at primary surgery. These figures are at least comparable with the response and progression-free survival data seen in large international randomised trials of platinum–taxane combinations.

The median progression-free survival was 19.5 months (95% CI: 13.4–25.6 months), which, despite the median duration of follow-up being 28 months, should be fairly accurate as more than half the total patients have progressed. In the SCOTROC II trial in which 500 patients received conventional paclitaxel/carboplatin therapy, the median progression-free survival was 15 months (Vasey, 2001, 2002). Thus, the progression-free survival reported here is encouraging, given both trials enrolled similar patient populations.

Gemcitabine, platinum, and paclitaxel as a triplet combination for first-line therapy of ovarian cancer has been developed by Hansen and colleagues (Hansen *et al*, 1999; Hansen, 2002). Patients with recurrent, pretreated disease were given cisplatin 75 mg m^−2^ (on day 1), paclitaxel 175 mg m^−2^ (on day 1), and gemcitabine 1000 mg m^−2^ (on days 1 and 8), whereas untreated patients (and relapsed patients demonstrating severe toxicity with the former regimen) were given carboplatin AUC 5 (on day 1), paclitaxel 175 mg m^−2^ (on day 1) and gemcitabine 800 mg m^−2^ (on days 1 and 8). The extremely promising overall response rate of 100% in 25 evaluable patients (radiological and/or CA 125) occurred at the expense of considerable haematologic toxicity.

In our trial, the incidence of infection, sensory neuropathy, and alopecia was low; however, the rate of pulmonary toxicity with weekly gemcitabine/paclitaxel therapy was of concern with a pattern of breathlessness, interstitial lung changes, and decreased DLCO in several patients. The symptoms were generally mild and reversible. As this pattern was recognised during the course of the study, it is not possible to determine if there were any predisposing factors such as smoking, or pre-existing respiratory disease.

Trials in non-small-cell lung cancer of weekly taxane/gemcitabine combinations have also identified this toxicity although it is very difficult to accurately identify pulmonary toxicity in this patient group. Bhatia *et al* conducted a phase II trial of weekly gemcitabine and paclitaxel in previously untreated patients. Gemcitabine 1000 mg m^−2^ and paclitaxel 110 mg m^−2^ were given on days 1, 8, and 15 every 28 days. Four patients (10%) developed hypoxia with interstitial lung shadowing on chest X-ray, and one patient died (Bhatia *et al*, 2002). Thomas *et al* also reported pneumonitis as the dose-limiting toxicity in a phase I/II trial of weekly gemcitabine and paclitaxel, and the trial was stopped after enrolling 12 patients as four patients experienced this toxicity (Thomas *et al*, 2000). In the SGCTG trial that was running concurrently in the United Kingdom, a similar phenomenon was seen in the patients who received the weekly docetaxel/gemcitabine (Vasey *et al*, 2003). Thus, it would appear that this toxicity might be particularly prevalent in the taxane/gemcitabine weekly schedules.

In summary, the approach of using sequential carboplatin followed by paclitaxel/gemcitabine is feasible, and the efficacy data are encouraging; however, the incidence of pulmonary toxicity is of some concern and may limit the use of this weekly regimen. Thus, the SGCTG are currently proposing to evaluate sequential carboplatin followed by the combination of gemcitabine/paclitaxel, but with a more conventional 3-week taxane dosing.
